# Gold Nanorod Integrated Electrochemical Sensing for Hyperglycaemia on Interdigitated Electrode

**DOI:** 10.1155/2019/9726967

**Published:** 2019-07-15

**Authors:** Shumin Zheng, Hong Zhang, Thangavel Lakshmipriya, Subash C. B. Gopinath, Na Yang

**Affiliations:** ^1^Department of Obstetrics, Dezhou People's Hospital, No. 1166 Dongfanghong West Road, Dezhou, Shandong Province, 253014, China; ^2^Institute of Nano Electronic Engineering, Universiti Malaysia Perlis, 01000 Kangar, Perlis, Malaysia; ^3^School of Bioprocess Engineering, Universiti Malaysia Perlis, 02600 Arau, Perlis, Malaysia

## Abstract

Gestational diabetes (hyperglycaemia) is an elevated blood sugar level diagnosed during the period of pregnancy and affects the baby's health. Hyperglycaemia has been found within the gestational weeks between 24 and 28, and the foetus has also the possibility of getting out prior to this test frame; it causes excessive birth weight, early birth, low-blood sugar level, respiratory distress syndrome, and type-2 diabetes to the mother. It creates a mandatory situation to identify the hyperglycaemia at least during the pregnancy weeks from 18 to 20. Further, a continuous monitoring of the level of glucose is necessary for the proper delivery. In this work, a method is introduced for glucose detection at 0.06 mg/mL, assisted by gold nanorod (GNR)-conjugated glucose oxidase (GOx) on interdigitated electrode sensor. In the absence of GNR, GOx shows the limit of glucose detection to be 0.25 mg/mL. Moreover, with GOx-GNR the reactions of all the glucose concentrations have recorded higher levels of the current from the baseline. With the specificity analysis, it was found that the glucose only reacts with GOx-GNR and discriminates other sugars efficiently. This method of detection is useful to diagnose and continuously monitor the glucose level during the pregnancy period.

## 1. Introduction

 Gestational diabetes (hyperglycaemia) occurs during the period of pregnancy with the elevated blood sugar level [1, 2]. During the period of pregnancy, higher levels of hormones are produced by the placenta. Those hormones impair the action of insulin in the cells and increase the level of blood sugar. As baby grows, higher insulin is counteracting the hormones produced by the placenta and provokes the increased blood sugar level, ultimately affecting the growth of the baby. In most cases gestational diabetes develops at the period of 20th week or during the late half of pregnancy [3–5]. Due to the variations in the glucose level during the period of pregnancy, generating an easier monitoring system is mandatory for a healthier pregnancy. Different sensing methods have been used to detect the level of glucose, and still researchers are working towards generating an efficient detection system. It is due to the potential issues with hyperglycaemia, and it affects the body systems including, heart, kidney, eye, and nerves. In this work, authors demonstrated the gold nanorod (GNR) integrated interdigitated electrode (IDE) to detect the level of glucose.

Nanomaterial assistance in the field of the biosensor is appreciated due to its ability to promote the high-sensitive detection with the lower signal-to-noise ratio. Various nanomaterials such as gold, silver, iron, platinum, titanium, palladium, and graphene have been used for the physical modification with the sensing surfaces or for increasing the surface area with the probe or analyte attachment in order to improve the detection limit [6–8]. Among different nanomaterials, gold is one of the excellent metals, being used in various sensors for detecting different diseases such as cancer, bacterial infection, nephrotoxicity, and viral infection. Moreover, due to its stability and optical properties gold is also used for imaging to target the diseases [9, 10]. Gold nanoparticle (GNP) conjugated probe molecules are readily proved in improving the sensitivity on the analyte detection [11, 12]. Lakshmipriya et al. used the GNP-conjugated streptavidin to detect the lower abundance of blood clotting protein, Factor IX in the human serum, and reached the detection at 100 pM [13]. In another research, GNP-conjugated antibody was used to detect the intact influenza virus at the lower count [14]. In addition, GNP has been efficiently utilized in the colorimetry-based assays against a wide range targets. Due to its color changing property in the presence of mono- or divalent salts, aggregation (red to purple) and dispersion (purple to red) strategies were utilized [15, 16]. GNP-conjugated aptamer or antibody is commonly used to detect different biomolecules such as DNA, RNA, and protein with simple bare-eye detection. The current study conjugated the GNR with glucose oxidase (GOx) to quantify the level of glucose with the help of interdigitated electrode (IDE) sensor [17, 18]. IDE sensor is the simple electrochemical system, proving as one of the efficient sensor to detect various kinds of analyte molecules, such as DNA, RNA, protein, enzyme, hormone, intact bacteria, and virus [19, 20]. Due to its sensitive current changes with the electrochemical sensor, the lower level of limit of detection has been reached with various targets [21–23]. In this work, we immobilized the GNR-GOx conjugation on the IDE sensing surface through 1,1′-carbonyldiimidazole (CDI) cross-linker and efficiently detected the level of glucose by monitoring the changes in the electrical current with the reaction of GOx.

## 2. Materials and Methods

### 2.1. Reagents and Biomolecules

Glucose oxidase (GOx), glucose, CDI, and Phosphate Buffer Saline (PBS) were purchased from Sigma-Aldrich (USA). GNR was from Nanocs, USA. Ethanolamine was bought from Fisher Scientific (UK). The control analytes (lactose and fructose) were obtained from Sigma-Aldrich (USA).

### 2.2. IDE Fabrication

IDE fabrication was followed as previously using different parameters for the physical modifications on the surface [20]. Upon completion of oxidation of the silicon wafers at high-temperature, the etching process was carried out using the aluminium. After the etching, other processes with developing were carried out similar to the previous procedure [20]. Lastly, before being proceeded with the zinc oxide attachment, the sensing surface was washed by 1 M potassium hydroxide with pH 9.0.

### 2.3. Immobilization of GOx on IDE Surface

The IDE sensing surface was immobilized with GOx to detect and quantify the level of glucose. For that, initially the IDE surface was modified by CDI at 0.5 M and was diluted in 100 mL of 30% acetone and dropped on the IDE surface and kept for 3 h at room temperature (RT). After that, the surface was washed using distilled water for three times, followed by reaction with 1 *μ*M of GOx (diluted in 10 mM PBS, pH 7.4) for 30 min at RT, and then the surface was washed again using PBS buffer. Then GOx-modified IDE surface was blocked by dropping 1 M of ethanolamine and kept for 30 min at RT. Finally, 1 mg/mL of glucose was added on the surface and the changes in the current level from the baseline were recorded. These changes in current were indicating the reaction of GOx and glucose.

### 2.4. GNR-GOx Conjugation and Stability Assay

To improve the detection, the prepared probe (GOx) was conjugated with GNR. A 1 *μ*M of GOx was mixed with 10 *μ*l of GNR (1 optical density) and kept for 30 min at RT. After the electrostatic conjugation, it was washed three times with distilled water and separated by centrifugation (10,000 x g for 5 min). The stability of this conjugation was confirmed by the salt-induced aggregation. For that, 500 mM of NaCl was added to only GNR and GNR-GOx conjugation and waited 10 min for the color changes from the red.

### 2.5. Glucose Detection on IDE Sensor in the Presence/Absence of GNR

Initially, 1 mg/mL of glucose was detected on the IDE sensing surface with two different probe conditions, such as GOx and GNR-GOx. For this reaction, the diluted 1 mg/mL of glucose was dropped on the GOx or GNR-GOx modified surfaces after the blocking step by ethanolamine. The differences in the current level were noted. With the above optimal conditions, to monitor the limit of detection, different concentrations of glucose (from 0.06 to 1 mg/mL) were reacted on both GOx and GNR-GOx modified surfaces.

### 2.6. Specific Detection of Glucose on IDE Surface

To check the specific detection of glucose on GNR-GOx modified IDE sensing surfaces, two different control experiments (lactose and fructose) were carried out and compared with the similar concentration of glucose (1 mg/mL). Upon addition of these analytes, the changes in the current level were recorded.

## 3. Results and Discussion

**Figure 1:**
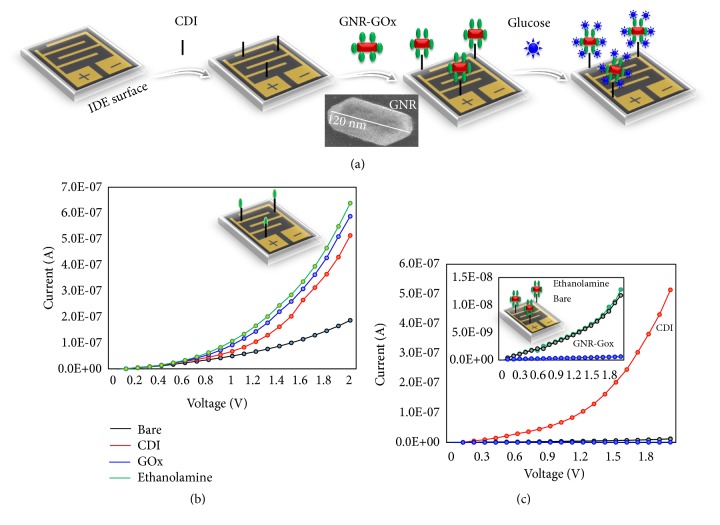
(a) Schematic representation of glucose detection on GNR-GOx modified IDE surface. GNR-GOx immobilized on the IDE surface through the cross-linker, CDI, and then glucose was detected by measuring the changes with the electrical signal. Probe immobilization on the IDE surfaces with (b) GOx immobilization on CDI-modified IDE surface. The surface was blocked with ethanolamine. With each immobilization the current output was increased. (c) GNR-GOx immobilization on CDI-modified IDE surface. The surface was blocked with ethanolamine. With each immobilization the current output was increased. The figure inset shows the illustration on IDE.

Identification followed by the quantification of the level of blood glucose is mandatory during the period of pregnancy [24, 25]. A higher level of glucose is causing various health issues to the mother and foetus during and after the pregnancy period and generates the complications. In addition, the elevated glucose level affects different parts in the physiological system, including heart, eyes, and kidney [26–28]. Different techniques and sensing strategies have been demonstrated to monitor the level of glucose. Further, researches have been carried out in high-performance manner with the assistance of gold nanomaterials [8, 29, 30]. In this work, an interdigitated electrode (IDE) sensor is used to quantify the level of glucose, to be suitable for the hyperglycaemia and enhance the sensitive detection by gold nanorod (GNR) conjugation.  Figure 1(a) explains the schematic representation for the detection and quantification of glucose on the IDE sensor. As shown in the figure, GNR-GOx conjugated probe was immobilized on IDE surface through the cross-linker (CDI), and then the glucose was allowed to react with GOx and quantified the level. It has been previously proved that gold nanomaterial conjugated probe was able to enhance the limit of detection. Since a larger number of the probes can attach on a single nanoparticle, there is a possibility of interacting a higher amount of target molecule [13, 29–31]. Since GOx can easily immobilize with GNR through the electrostatic interaction, in this work GOx was immobilized on the surface of nanoscaled GNR (~120 nm x 25 nm) and enhanced the detection level. In addition, the proper orientation of probe on the sensing surface is playing a vital role to interact with the higher number of targets [32–34]. In general, biomolecular attachment on the sensing surface is not expected to be 100%. In the current research, due to this reason, the attachment of GOx is not uniform. Even though the same phenomena are applied on the GNR for conjugating GOx through amine groups, due to the creation of more space by the GNR, higher number of GOx attached and reacted with glucose with a higher signal enhancement.

### 3.1. GOx and GNR-GOx Probe Immobilization on IDE Surface

Figures 1(b) and 1(c) show GOx probe immobilization on the IDE surface with (Figure 1(b)) and without GNR (Figure 1(c)). To capture GOx, CDI has been used; it is a highly reactive carboxylating agent, exhibiting two acyl imidazole leaving groups and forming the reactive carbonyl groups on the hydroxyl sensing surface. This surface will couple the amino group on the GOx to the hydroxyl group via an amide linkage. In Figure 1(b), the bare line shows the maximum current level as 1.87 E-07, and after modifying the surface by CDI it altered the current level to 5.14 E-07, confirming the CDI modification. When GOx was attached to the CDI surface, the level of the current was changed from 5.14 E-07 to 5.88 E-07. The remaining unattached areas on the IDE were blocked by ethanolamine, confirmed by increasing the current level to 6.38 E-07. All these proper increments are showing that the probe, GOx, is immobilized on the IDE surface and ready to detect the level of glucose. Similar method was used to immobilize the GNR-GOx probe on IDE surface. Figure 1(c) shows the step-by-step immobilization and the proportional changes in the current. Bare line shows the basic current at 1.28 E-08 and after adding the CDI it increased to 5.14 E-07. When adding the GNR-GOx probe, the current level was drastically reduced to 1.18 E-08, due to the surface charge on the GNR. After that, ethanolamine was added to cover the remaining free-sites on the IDE surface. In this case, the ethanolamine shows only the small variation of the current compared to the previous case, which is due to the larger surface occupied by the GNR-GOx.

### 3.2. Comparison of Glucose Detection on GOx and GNR-GOx Modified Surfaces

**Figure 2:**
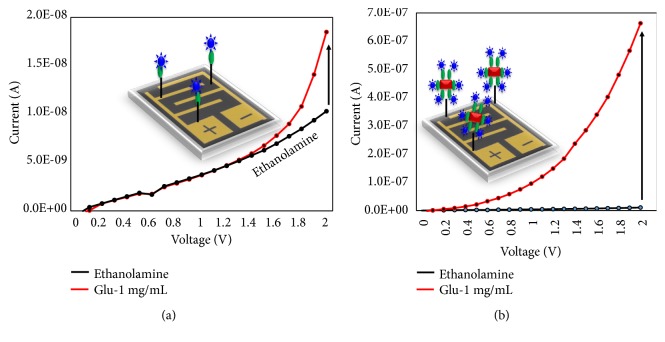
Detection of glucose (1 mg/mL) on (a) GOx modified surface and (b) GNR-GOx modified surface. The spectral changes are shown. The figure inset shows the illustration on IDE.

At the beginning to validate 1 mg/mL of glucose was tested on the GOx and GNR-GOx modified surfaces (Figures 2(a) and 2(b)). As shown in Figure 2(a), the current increment was noticed from 1.03 E-08 to 1.85 E-08 (difference is 8.2 E-09), after adding 1 mg/mL of glucose on GOx modified surface. At the same time, the current level was changed from 1.18 E-08 to 6.54E-07 (the difference is 6.4 E-07) on the GNR-GOx modified surface. This is ~80 times higher current change compared to the condition without GNR. This increment in current change is due to the higher number of GNR-GOx attachment on the surface of the IDE.

### 3.3. Limit of Detection on GOx and GNR-GOx Modified Surfaces

**Figure 3:**
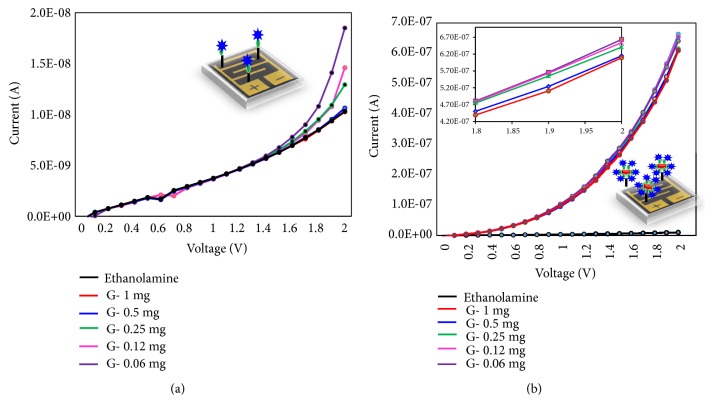
Limit of glucose detection on (a) GOx modified surface and (b) GNR-GOx modified surface. Glucose was titrated from 0.06 to 1 mg/mL. The enlarged portion is shown as inset. The figure inset shows the illustration on IDE.

**Figure 4:**
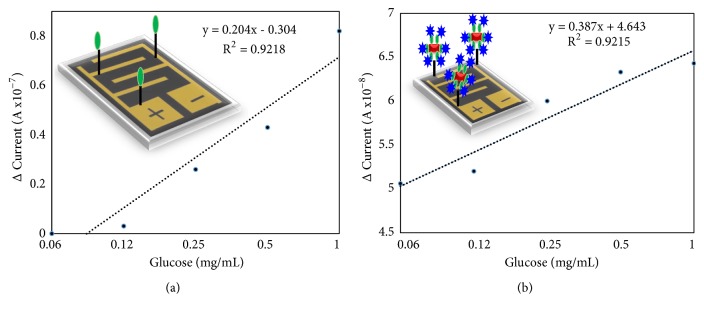
Linear graph on the limit of detection, with (a) GOx modified surface and (b) GNR-GOx modified surface. The figure inset shows the illustration on IDE.

After preliminary comparison of the glucose detection on GOx and GNR-GOx modified surfaces, the limit of detection was also compared on these modified surfaces. Since it was proved that GNR-GOx modified surface exhibits higher current increment, it was expected to detect glucose at the lower level. For that, we titrated the glucose concentrations from 0.06 to 1 mg/mL and tested on both GOx and GNR-GOx modified surfaces.  Figure 3(a) shows the limit of detection of glucose on GOx-IDE surface, it was found that the concentration of glucose at 0.06 and 0.12 mg/mL did not show any changes of current from the baseline, which means there is a very lower level of glucose that cannot interact with GOx-modified surface. After adding 0.25 mg/ mL of glucose, increment in the current level from 1.03E-08 to 1.29 E-07 was noticed. Then with increasing glucose concentration further, the current was gradually increased. At the concentration of 0.5 and 1 mg/mL, the curves show the changes with 1.46E-08 and 1.85 E-08, respectively. In conclusion, the limit of glucose detection was found as 0.25 mg/mL on GOx-modified surface. The similar glucose concentrations were titrated on GNR-GOx modified surface. It was found that even at the concentration of 0.06 mg/mL, there is a clear change in current from 1.18E-08 to 6.09 E-07. With increasing the glucose concentration to 0.12 mg/mL (6.1-07), 0.25 mg/mL (6.15E-07), 0.5 mg/mL (6.41E-07), and 1 mg/mL (6.54E-07), the respective current increment levels were noted (Figure 3(b)). Figures 4(a) and 4(b) show the linear graph of the limit of glucose detection on GOx and GNR-GOx modified surfaces. This result clearly showed that glucose can be detected below the range of 0.06 mg/mL on the GNR-GOx modified IDE surface with the estimation based on 3*σ*. In Figure 4, with only GOx the current changes are low with all the concentrations of glucose; at the same time, GNR-GOx shows a big difference with all the concentrations of glucose tested. Even lower concentrations are possible to detect with this sensing method. Apparently, the detected glucose range is possible to implement with the normal level of glucose detection in the blood (7.2 to 9.9 mg/ml). Currently, our research is also focusing to detect glucose in the saliva using the similar sensing strategy and is considered promising.

### 3.4. Specific Detection of Glucose on GNR-GOx Modified IDE Surface

**Figure 5:**
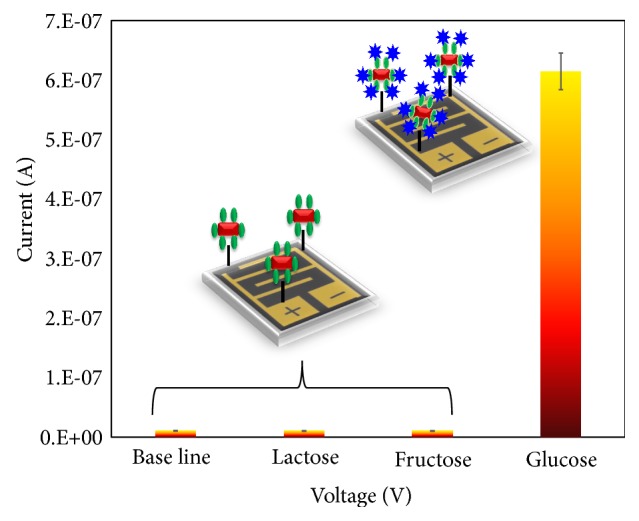
Specific detection of glucose on GNR-GOx modified IDE surface. Control experiments were carried out using lactose and fructose and compared with specific 1 mg/mL of glucose detection. The figure inset shows the illustration on IDE.

To evaluate the specific detection of glucose on GNR-GOx modified IDE sensing surface, two different control experiments on lactose and fructose were carried out and compared with the specific glucose detection at 1 mg/mL. As shown in Figure 5, the control experiment did not show any significant current changes, at the same time 1 mg/mL of glucose showed an apparent increment in the current from the baseline. From this result, it was concluded that glucose was detected specifically on the GNR-GOx modified IDE surface without any biofouling.

Due to the surface charge of the biomolecules, the electronic signal will be varied either by the current increment or decrement. Further, depending on the biomolecular interaction on the IDE surface, the difference in the dipole moment and molecular vibration occurs between the dielectrode [20]. The dipole moment is higher while binding strength exhibits between GOx and glucose, ultimately the creation of the local structure has triggered the vibrations. These efficient charge displacements are leading the electrical current changes with the concentration increments [20]. In the current study, when GOx binds on the surface, the charge was changed with the ultimate alteration in the current. On the other hand, GNR-GOx bounded abundantly on the IDE surface and could observe huge changes in the current level. The reaction of GOx with the substrate (glucose) was measured specifically without the interference from other chemical reactions, such as GOx or GNR-GOx attachment on CDI. This specific change was anchored by the analysis shown in Figure 5.

## 4. Conclusion

Monitoring hyperglycaemia is mandatory for the healthy mother and the foetus. A convenient and easier method of glucose detection strategy was carried out on the IDE electrochemical sensor, assisted with gold nanorod conjugated glucose oxidase (GNR-GOx). The limit of deletion of glucose on the surface with only GOx was found as 0.25 mg/mL, whereas GNR-GOx modified IDE surface has displayed a lower limit of detection as 0.06 mg/mL and also showed a great increment of current in all the concentrations of glucose tested compared to the results obtained from only GOx-modified surface. Moreover, the specific reaction was carried out with two different control experiments; it was found that GNR-GOx modified surface only recognized the glucose. This result indicates that the glucose was specifically detected on the chemically modified IDE sensing surface.

## Data Availability

The data used to support the findings of this study are available from the corresponding author upon request.
